# Corneal topography in preoperative evaluation for laser keratorefractive surgery – a review

**DOI:** 10.22336/rjo.2020.55

**Published:** 2020

**Authors:** Bogdana Tăbăcaru, Tudor Horia Stanca

**Affiliations:** * “Prof. Dr. Agrippa Ionescu” Clinical Emergency Hospital, Bucharest, Romania; “Carol Davila” University of Medicine and Pharmacy, Bucharest, Romania

**Keywords:** corneal astigmatism, corneal ectasia, corneal topography, preoperative evaluation, refractive surgery

## Abstract

Corneal topography is a mandatory investigation in the preoperative evaluation of the patient candidate for laser keratorefractive surgery, in order to assess the corneal shape, to determine the radii of curvature and the corneal thickness. Abnormal corneal topography is the most important identifiable risk factor for corneal ectasia. This paper reviews the principles of successive generations of topographers and illustrates several normal and abnormal corneal topographies.

## Introduction

Laser keratorefractive surgery has numerous contraindications, both systemically and locally, of which the corneal ectatic disorders are the most important to recognize at the preoperative evaluation in order to exclude these cases from surgery [**1**-**3**]. Therefore, corneal topography is probably the most important investigation in preoperative screening and should be performed in all patients addressed for laser refractive surgery [**1**,**2**]. Corneal topography also plays a key role in postoperative evaluation of patients with unexpected results after refractive surgery [**2**,**4**].

## Review

**Current technologies of corneal topography**

The term “topography” is derived from the Greek words “topos” (place) and “graphein” (to write) and refers to the study and representation of forms, being used especially in geography and astronomy [**1**,**5**,**6**]. In ophthalmology, the term “corneal topography” is improperly used in order to characterize the shape of the corneal surface and its radii of curvature [**1**,**5**,**6**]. First-generation corneal topographers are devices that project a system of concentric light circles (the Placido disk) onto the corneal surface, measure their angle of reflection and thus calculate the corneal curvature at various points, providing information on the corneal shape [**1**,**6**]. There are several generations of corneal topographers, according to the technology they use. The Placido disc method is the most widely used [**1**,**7**,**8**]. Second-generation corneal tomographers, Orbscan® type (Bausch and Lomb, USA), assesses the corneal elevation through optical sections obtained by combining Placido disc and slit scanning technologies, thus being able to characterize the posterior face of the cornea [**1**,**6**-**8**].

Corneal tomographers are new (third) generation devices, their technology allowing digital reconstruction of the structures of the anterior segment, without using a curvature system [**1**,**6**]. The term “tomography” is also derived from the Greek words “tomos” (section) and “graphein” (to write) [**1**,**6**]. The technology of corneal tomographers uses a Scheimpflug type rotating camera that allows the analysis of both anterior and posterior faces of the cornea, by direct measurements, not only by mathematical assessments as in the case of topographers [**1**,**7**,**8**]. In contrast, Scheimpflug technology provides less information on possible distortion of the anterior corneal face compared to the Placido disc [**1**,**9**].

Modern capture and analysis systems that combine the Scheimpflug rotating camera, the Placido disk and the slit scanning, such as the Galilei® (Ziemer, Switzerland) and Sirius® (Schwind Eye-Tech-Solutions, Germany), were created to combine the advantages of topographers and tomographers [**1**,**10**].

**Corneal topographic analysis**

Corneal topographic assessment frequently involves the analysis of four maps: the pachymetric map, the anterior elevation map, the posterior elevation map and a map of corneal curvature [**1**,**11**,**12**]. Compared to ultrasound pachymetry, the topographical systems provide a thicker value of the corneal thickness, as the anatomical limits for measurements are different [**1**,**11**]. However, even if the topographers measure the cornea from the tear film layer to the Descemet’s membrane, the integrated computer can replicate the ultrasound pachymetry values using an adjustment factor of 0.92, the so-called “acoustic factor” [**1**,**11**].

The elevation maps are obtained by comparing the anterior and posterior corneal surface to a best fitted surface, usually to a sphere (“Best-fit Sphere”) and recording every difference between both surfaces [**1**,**11**,**13**]. For both corneal surfaces, the analysis of the elevation maps provides valuable data regarding: the corneal apex, the thinnest corneal point and the center of the corneal central region [**13**]. The posterior corneal surface is very useful for the diagnosis of the preoperative and postoperative keratectasia as it is not altered by the hyperplastic effect of corneal epithelium nor by the corneal surgical maneuvers as the corneal flap creation or the excimer laser ablation [**13**].

There are more principles for determining corneal curvature maps: axial map (in sagittal section) or tangential map (also known as meridional map or instantaneous radius of curvature) [**1**,**5**]. For the axial map, the corneal surface is considered to have spherical geometry, consideration that is erroneous [**13**]. For this reason, the true shape and the power of the peripheral area of the cornea are not properly assessed [**13**]. The axial map is useful for qualitative analysis of the anterior cornea [**1**,**5**] and its interpretation is easy for less-experienced users [**13**]. The tangential map represents the curvature of the corneal periphery [**13**] more accurately and provides a more detailed analysis of changes of the anterior corneal surface [**1**,**5**]. The corneal surface is reconstructed based on local curvature radii. However, the interpretation of the tangential map is more difficult and more complex than in the case of the axial map [**13**]. 

**Topography of normal cornea**

There is a wide range of normal topographical aspects, the cornea not being perfectly regular like the calibration spheres [**1**,**11**]. Physiologically, in childhood and adolescence an up to 0.75 D with-the-rule astigmatism may be present as a result of the slight pressure of the upper eyelid on the cornea [**1**,**11**]. Towards senescence the astigmatism turns against-the-rule, after a period in which the cornea acquires a more spherical shape [**1**,**11**]. 

The normal cornea is prolate, which means that it is steep in the center and flat in the periphery [**1**,**11**]. When this aspect is compared with a best-fit sphere as reference level, the central cornea is steeper and the mid-periphery is flatter [**1**,**11**]. On corneal topographic assessment, cold colors (blue hues) encode flattened corneal areas and warm colors (red hues) encode prominent ones [**1**,**7**,**14**].

There are several characteristics of normal corneas. The nasal corneal half is flatter than the temporal one [**1**,**11**,**15**]. This aspect will be highlighted on the topographic maps as a steeper slope toward blue hues in the nasal cornea [**1**,**11**,**15**]. An asymmetry between the upper and lower halves of the cornea is also found [**1**,**15**]. The steepest point is usually located superotemporal to the visual axis while the thinnest point is located in the inferotemporal quadrant [**1**,**15**].

**Fig. 1:**
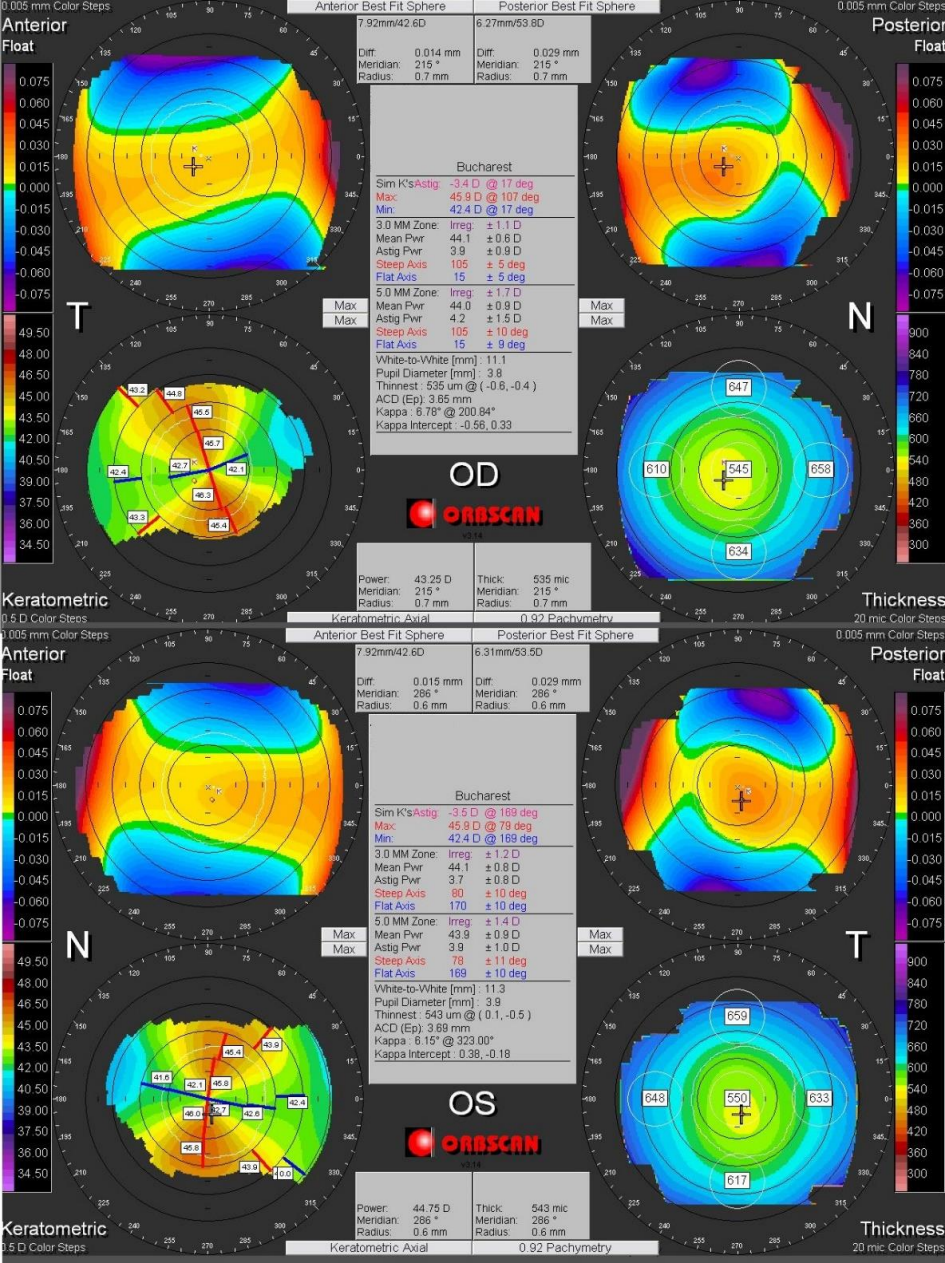
Corneal topography demonstrating mirror topographic images (enantiomorphic symmetry) of both eyes of the same patient

In the normal eyes, the two corneas of the same patient are very similar, both eyes tending to have mirror topographic images (enantiomorphic symmetry) (**Fig. 1**) [**1**,**11**,**15**].

In preoperative evaluation for corneal refractive surgery, special attention is paid to posterior corneal surface, which is considered to be normal if two features of greatest importance are gathered together [**1**,**11**]. The steepest point of the posterior corneal surface should be located centrally and not away from the center in an area of corneal thinning [**1**,**11**]. Also, the difference between the steepest and the flattest points of the posterior corneal surface should not exceed 50 µm [**1**,**11**].

**Fig. 2:**
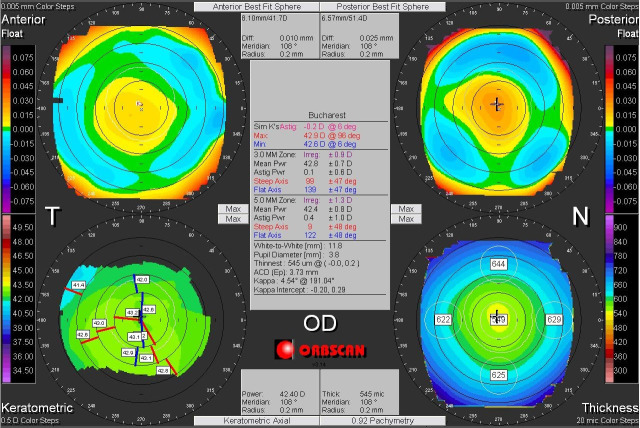
Corneal topography of a non-astigmatic cornea (simulated cylinder = 0.2 D). Reprinted from “Femtosecond Laser – Excimer Laser Platform for ametropias surgery (PhD thesis)”, by Tăbăcaru B, 2019, “Carol Davila” University of Medicine and Pharmacy, Bucharest, Romania

**Normally**, the result for a nonastigmatic cornea will highlight on the topographic map a relatively uniform color in the center and a slight flattening towards the periphery (**Fig. 2**) [**1**,**2**].

**Fig. 3:**
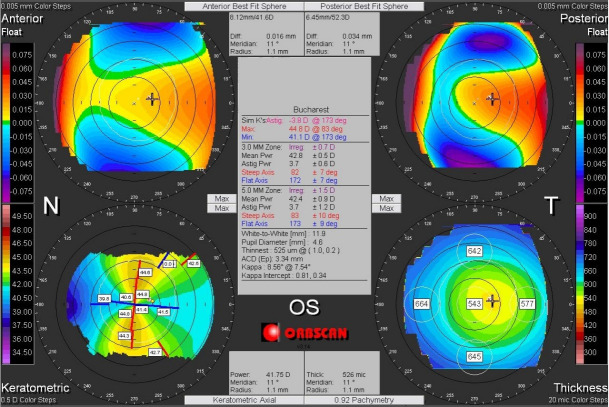
Corneal topography of a regular with-the-rule astigmatism (simulated cylinder = 3.8 D, steepest meridian at 83o). Reprinted from “Femtosecond Laser – Excimer Laser Platform for ametropias surgery (PhD thesis)”, by Tăbăcaru B, 2019, “Carol Davila” University of Medicine and Pharmacy, Bucharest, Romania

**Fig. 4:**
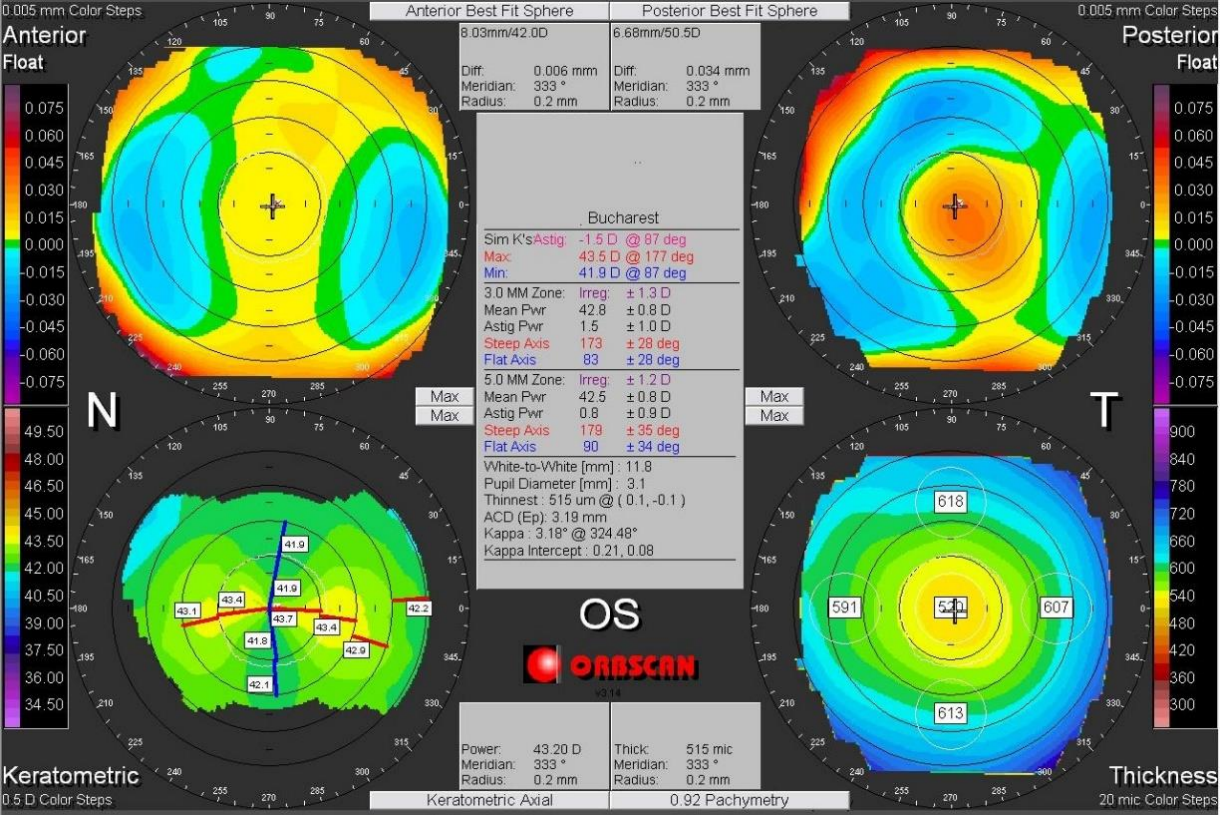
Corneal topography of a regular against-the-rule astigmatism (simulated cylinder = 1.5 D, steepest meridian at 177o). Reprinted from “Femtosecond Laser – Excimer Laser Platform for ametropias surgery (PhD thesis)”, by Tăbăcaru B, 2019, “Carol Davila” University of Medicine and Pharmacy, Bucharest, Romania

**Fig. 5:**
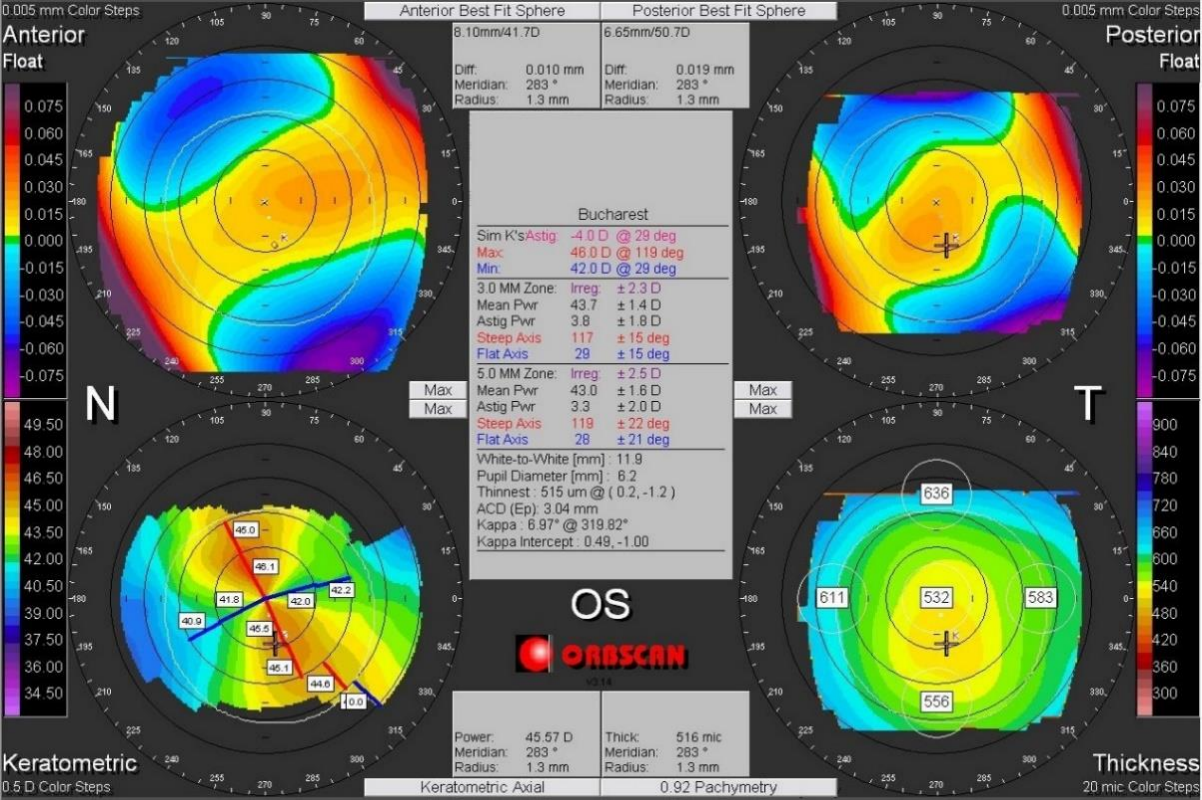
Corneal topography of a regular oblique astigmatism (simulated cylinder = 4.0 D, steepest meridian at 119o). Reprinted from “Femtosecond Laser – Excimer Laser Platform for ametropias surgery (PhD thesis)”, by Tăbăcaru B, 2019, “Carol Davila” University of Medicine and Pharmacy, Bucharest, Romania

In **regular astigmatism**, corneal topography displays a symmetrical bow-tie pattern, the principal meridians (of greatest and least powers) being located 90 degrees apart [**1**,**11**]. In the with-the-rule astigmatism, which is the more common form of regular astigmatism, the steepest radius and the bow-tie pattern are located in the vertical meridian (**Fig. 3**) [**1**,**11**]. Less frequent types of regular astigmatism are the against-the-rule astigmatism, when the bow-tie pattern and the steepest meridian are located horizontally (**Fig. 4**), and the oblique astigmatism, when both the steepest meridian and the bow-tie pattern are diagonally placed (**Fig. 5**) [**1**,**11**].

**Topography of abnormal cornea**

**Irregular astigmatism** occurs when two halves of the cornea are consistently different (superior versus inferior or nasal versus temporal) [**1**,**11**]. The two sides of the bow-tie may differ in magnitude (asymmetrical bow-tie) or may not be orthogonally aligned to each other (skew of steepest radial axes) or both [**1**,**11**]. 

**Fig. 6:**
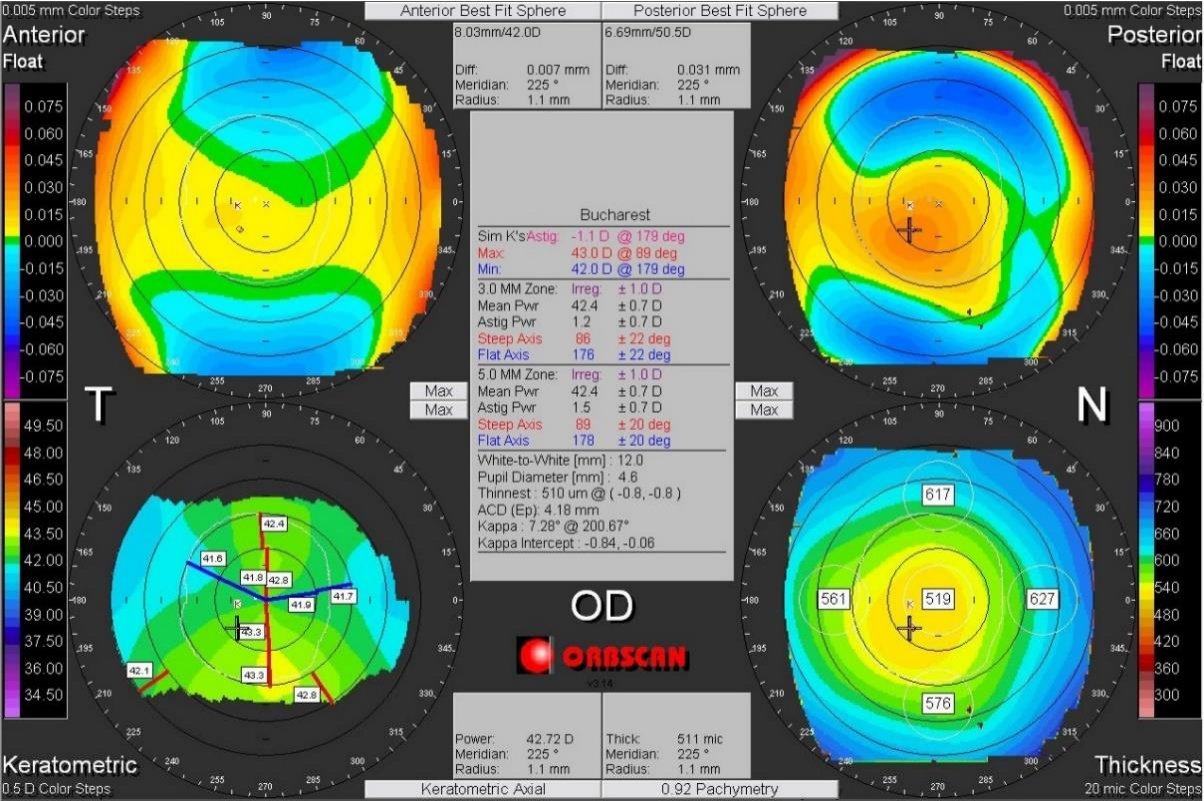
Corneal topography of an irregular astigmatism, with asymmetry between the superior and the inferior corneal halves, but with no skewing of the steepest meridians. Reprinted from “Femtosecond Laser – Excimer Laser Platform for ametropias surgery (PhD thesis)”, by Tăbăcaru B, 2019, “Carol Davila” University of Medicine and Pharmacy, Bucharest, Romania

**Fig. 7:**
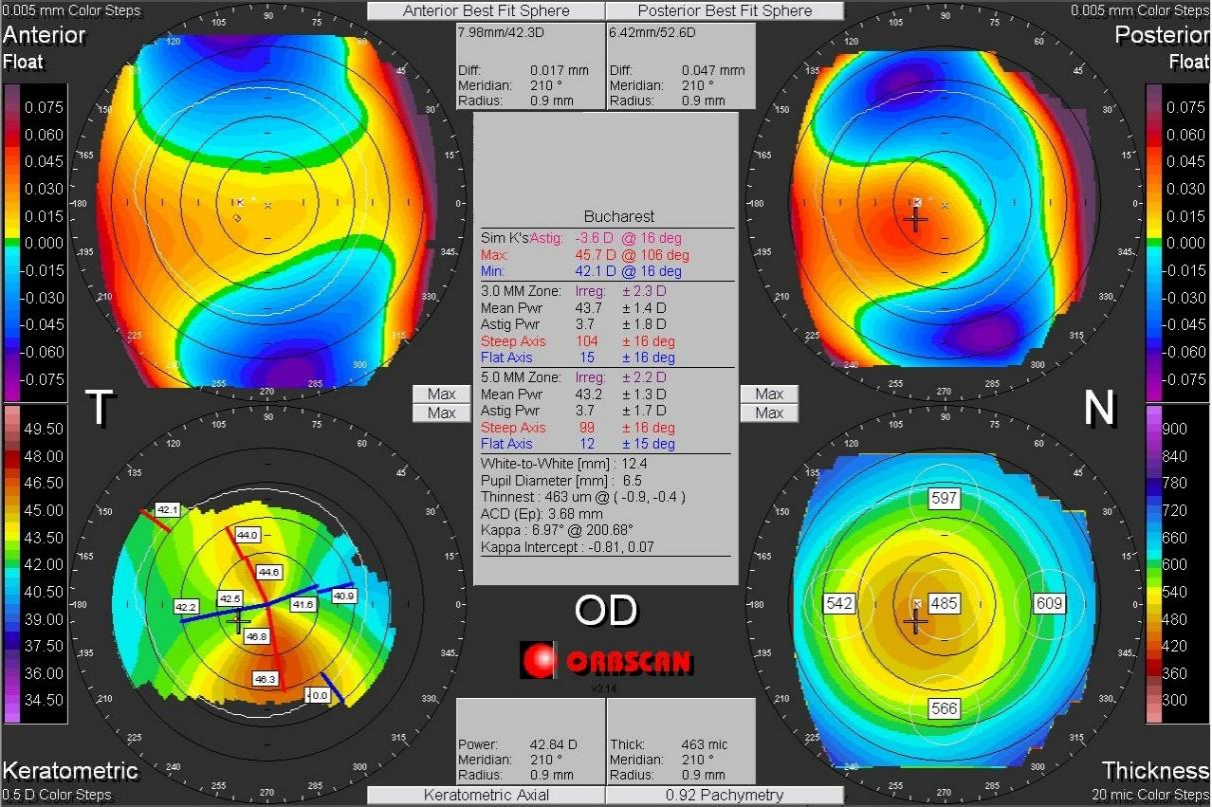
Corneal topography of an irregular astigmatism, with topographic changes consistent with keratoconus. Reprinted from “Femtosecond Laser – Excimer Laser Platform for ametropias surgery (PhD thesis)”, by Tăbăcaru B, 2019, “Carol Davila” University of Medicine and Pharmacy, Bucharest, Romania

**Fig. 8:**
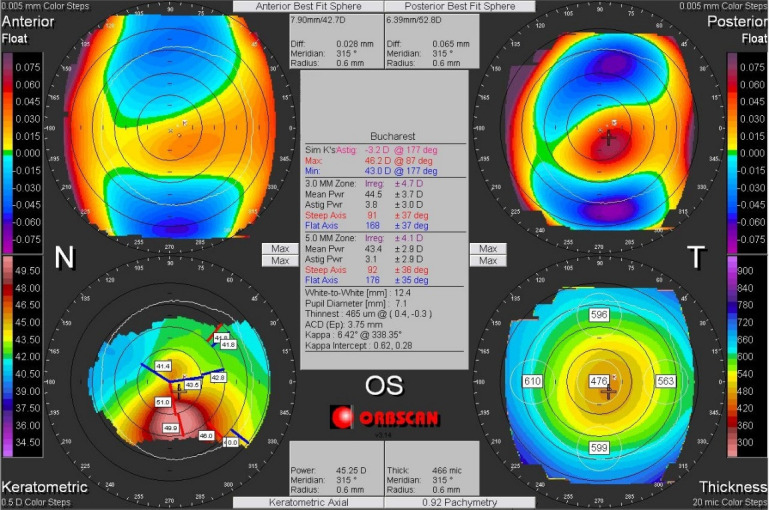
Corneal topography of an irregular astigmatism, with topographic changes consistent with keratoconus, also associated with clinical signs. Reprinted from “Femtosecond Laser – Excimer Laser Platform for ametropias surgery (PhD thesis)”, by Tăbăcaru B, 2019, “Carol Davila” University of Medicine and Pharmacy, Bucharest, Romania

**Fig. 9:**
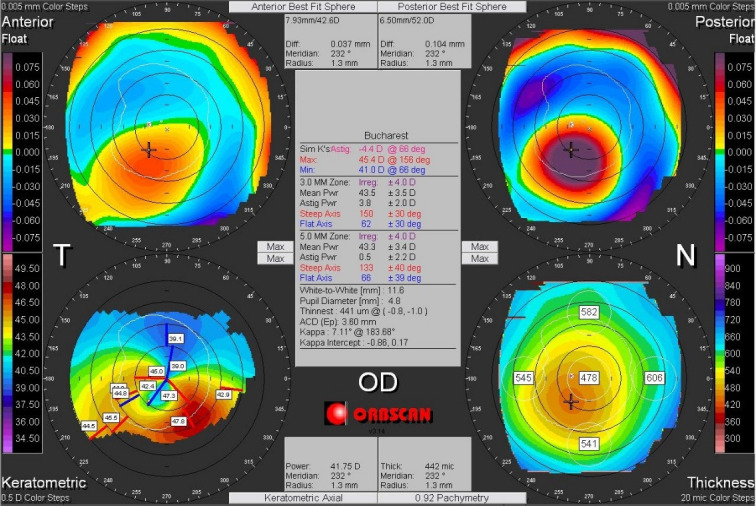
Corneal topography of an irregular astigmatism, with topographic changes consistent with Pellucid Marginal Degeneration. Reprinted from “Femtosecond Laser – Excimer Laser Platform for ametropias surgery (PhD thesis)”, by Tăbăcaru B, 2019, “Carol Davila” University of Medicine and Pharmacy, Bucharest, Romania

Corneal asymmetry does not always indicate the presence of a corneal pathology (**Fig. 6**) but its presence requires caution in keratorefractive laser treatment and topographic follow-up over a long period of time [**1**,**11**]. In other cases of irregular astigmatism, the corneal topography may highlight the presence of a subclinical keratoconus (**Fig. 7**), may confirm an obvious clinical keratoconus (**Fig. 8**) or may reveal another type of corneal ectasia as pellucid marginal degeneration (**Fig. 9**) [**1**,**2**,**11**].

**Indices of corneal irregularities**

In addition to generating corneal topographic maps, another important use of corneal topographers and tomographers in the preoperative evaluation is the assessment of various indices that allows quantification of the level of irregularity of the corneal morphology [**13**]. Depending on the analysis approach, the interpretation of the topographic corneal characteristics could be based on a single index (univariate quantitative analysis) or a combination of multiple indices (multivariate quantitative analysis) [**13**]. Some of the main indices assessed are: simulated keratometry, surface asymmetry index, surface regularity index, central keratometry, inferior-superior index, average corneal power, skew of the steepest radial axis, irregular astigmatism index, apex curvature, asphericity coefficient, etc. [**13**]. Each of these indices is highly specific for the corneal topographer for which it has been developed, the values obtained could not be extrapolated from a corneal topographer to another [**13**]. 

## Conclusions

The corneal topographic and tomographic evaluation is performed for each point of the corneal surface, thus, after being color-coded, the results are transposed into pachymetric, elevation and power maps of the cornea [**1**,**2**,**7**,**8**,**14**]. Both anterior and posterior corneal faces can be characterized this way [**1**,**2**,**7**,**8**,**14**]. Corneal topography and tomography are also useful for assessing the shape and degree of the astigmatism [**1**,**2**,**7**,**8**,**14**]. Abnormal corneal topography is the most important identifiable risk factor for corneal ectasia [**16**]. Corneal topography is a mandatory investigation in the preoperative evaluation of the patient candidate for laser keratorefractive surgery, in order to assess the corneal shape, to determine the radii of curvature and the corneal thickness [**1**,**2**,**7**,**8**,**14**].

**Acknowledgements**

Not applicable.

**Sources of Funding**

None.

**Disclosures**

None of the authors has any financial or proprietary interests to disclose.
